# Matrix Metalloproteinases in Chemoresistance: Regulatory Roles, Molecular Interactions, and Potential Inhibitors

**DOI:** 10.1155/2022/3249766

**Published:** 2022-05-09

**Authors:** Bernadette Xin Jie Tune, Maw Shin Sim, Chit Laa Poh, Rhanye Mac Guad, Choy Ker Woon, Iswar Hazarika, Anju Das, Subash C. B. Gopinath, Mariappan Rajan, Mahendran Sekar, Vetriselvan Subramaniyan, Neeraj Kumar Fuloria, Shivkanya Fuloria, Kalaivani Batumalaie, Yuan Seng Wu

**Affiliations:** ^1^Department of Pharmaceutical Life Sciences, Faculty of Pharmacy, Universiti Malaya, Kuala Lumpur 50603, Malaysia; ^2^Centre for Virus and Vaccine Research, School of Medical and Life Sciences, Sunway University, Selangor 47500, Malaysia; ^3^Department of Biomedical Science and Therapeutics, Faculty of Medicine and Health Science, Universiti Malaysia Sabah, Kota Kinabalu, 88400 Sabah, Malaysia; ^4^Department of Anatomy, Faculty of Medicine, Universiti Teknologi MARA, Sungai Buloh, 47000 Selangor, Malaysia; ^5^Department of Pharmacology, Girijananda Chowdhury Institute of Pharmaceutical Science, Guwahati 781017, India; ^6^Department of Pharmacology, Royal School of Pharmacy, Royal Global University, Guwahati 781035, India; ^7^Faculty of Chemical Engineering Technology, Universiti Malaysia Perlis (UniMAP), Arau, 02600 Perlis, Malaysia; ^8^Institute of Nano Electronic Engineering, Universiti Malaysia Perlis, Kangar, 01000 Perlis, Malaysia; ^9^Department of Natural Products Chemistry, School of Chemistry, Madurai Kamaraj University, Madurai 625021, India; ^10^Department of Pharmaceutical Chemistry, Faculty of Pharmacy and Health Sciences, Royal College of Medicine Perak, Universiti Kuala Lumpur, Ipoh 30450, Perak, Malaysia; ^11^Department of Pharmacology, School of Medicine, Faculty of Medicine, Bioscience and Nursing, MAHSA University, Selangor 42610, Malaysia; ^12^Faculty of Pharmacy, AIMST University, Semeling, Bedong, Kedah 08100, Malaysia; ^13^Department of Biomedical Sciences, Faculty of Health Sciences, Asia Metropolitan University, 81750 Johor Bahru, Malaysia; ^14^Department of Biological Sciences, School of Medical and Life Sciences, Sunway University, Selangor 47500, Malaysia

## Abstract

Cancer is one of the major causes of death worldwide. Its treatments usually fail when the tumor has become malignant and metastasized. Metastasis is a key source of cancer recurrence, which often leads to resistance towards chemotherapeutic agents. Hence, most cancer-related deaths are linked to the occurrence of chemoresistance. Although chemoresistance can emerge through a multitude of mechanisms, chemoresistance and metastasis share a similar pathway, which is an epithelial-to-mesenchymal transition (EMT). Matrix metalloproteinases (MMPs), a class of zinc and calcium-chelated enzymes, are found to be key players in driving cancer migration and metastasis through EMT induction. The aim of this review is to discuss the regulatory roles and associated molecular mechanisms of specific MMPs in regulating chemoresistance, particularly EMT initiation and resistance to apoptosis. A brief presentation on their potential diagnostic and prognostic values was also deciphered. It also aimed to describe existing MMP inhibitors and the potential of utilizing other strategies to inhibit MMPs to reduce chemoresistance, such as upstream inhibition of MMP expressions and MMP-responsive nanomaterials to deliver drugs as well as epigenetic regulations. Hence, manipulation of MMP expression can be a powerful tool to aid in treating patients with chemo-resistant cancers. However, much still needs to be done to bring the solution from bench to bedside.

## 1. Introduction

**Figure 1:**
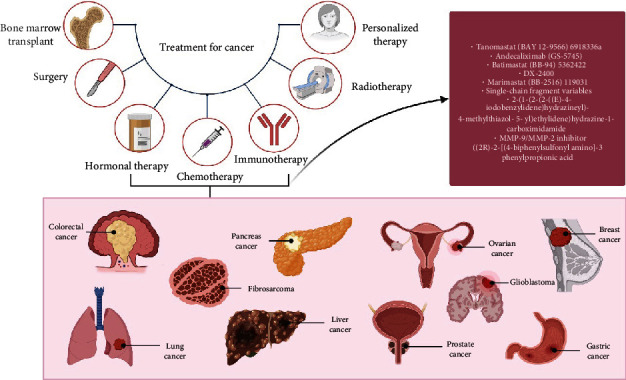
Types of cancer treatment. Different modalities evolving from conventional methods, such as surgery, chemotherapy, and radiotherapy, towards more personalized and precise therapies, including such as immunotherapy, hormonal therapy, and targeted therapy, have been used to treat various cancers. For targeted therapy, different inhibitors of MMPs have been or are testing preclinically and clinically due to their crucial roles in cancer progression and chemoresistance.

Cancer is one of the major causes of disease and death globally, accounting for 18.1 million new cases in 2018 [1]. Despite various efforts have been made in advancing treatment options and efficacy, the morbidity and mortality rate of cancer patients are still on the rise due to metastasis [2]. Treatment modalities for cancer include surgery, radiation therapy, and systemic treatment such as chemotherapy, targeted therapy, hormonal therapy, and immunotherapy (Figure 1) [3], of which, chemotherapy is the principal modality for cancer treatment [4]. It is used as a curative treatment for a small number of malignancies as well as having a palliative role for most metastatic epithelial malignancies and adjuvant role in several types of resected epithelial malignancies [5]. Chemotherapy activates the biochemical program involved in the cell replication and causes selective apoptosis via the production of reactive oxygen species as well as influencing the activities of relevant enzymes responsible for cell proliferation [6].

Most chemotherapeutic drugs target cell cycle machinery by relying on the differences in the frequency of cell division to differentiate between cancer and normal cells. Within this process, slow-growing cancer clones will survive and evolve into new fast-growing strains. Chemotherapy kills most susceptible cancer cells followed by sending tumors into remission for weeks or months, after which it recurs as a more aggressive organism [7, 8]. The more chemotherapy is given, the higher the aggressiveness of relapse. The biggest challenge in cancer management is the resistance to chemotherapeutic agents [9], which can be categorized into intrinsic or acquired resistance. Intrinsic resistance can be a result of innate characteristics of chemoresistance or activation of these pathways. For example, the most common intrinsic resistance arises from mutations of tumor suppressor genes such as p53 [10], which disable apoptosis in tumor cells, thereby increasing the population of chemo-resistant tumor cells. Acquired resistance can be categorized as the gradual reduction of anticancer drug efficacy. The most commonly reported acquired resistance is by overexpression of ATP-binding cassette (ABC) transporters that expel drug molecules out of cells via drug efflux [11]. Furthermore, benign tumor cells that turn into malignant also could acquire chemoresistance through invasion and metastasis by epithelial-to-mesenchymal transition (EMT) process [12]. EMT is responsible for the migration and invasion of cancer cells by transforming epithelial-like cells into a more aggressive mesenchymal phenotype, making them less sensitive to chemotherapeutic agents [13].

Due to increasing occurrences of drug resistance in cancer and the plethora of mechanisms exercised by cancerous cells to overcome and evade drug effects, novel molecular targets are constantly under investigation and development to resolve the issue. Reducing or inhibiting cancer metastasis may be the most universal option. One such method involves inhibiting or suppressing matrix metalloproteinase (MMP) activity. MMPs are a class of enzymes commonly observed in the degradation of the extracellular membrane (ECM), and they are critical in cancer metastasis, especially via their involvement in EMT and cancer migration [12]. MMPs promote the invasion of malignant cells through connective tissues and blood vessel walls by degrading the basement membrane and extracellular matrix, allowing cancerous cells to migrate and metastasize [14]. MMP expressions are tightly regulated and observed in low concentrations in normal conditions, while MMP overexpression is suggestive of cancer metastasis, progression, and poor prognosis [15]. The differential expression of MMPs between tumors and matched nontumors makes them a potential diagnostic and prognostic biomarker. Furthermore, due to their implied significance in cancer metastasis and chemoresistance, several MMP inhibitors have been developed to reduce their biological effects [16].

This review discusses and provides comprehensive insights into the biological and regulatory roles of MMPs that are commonly involved in chemoresistance and associated molecular mechanisms. Besides, it briefly discusses their diagnosis and prognosis values in cancer. It also describes the existing and potential MMP inhibitors as treatment options for chemoresistance as well as challenges faced in the development of MMP inhibitors.

## 2. Occurrence of Chemoresistance in Cancer

### 2.1. Chemoresistance

Chemoresistance is described as the ability of cancer cells to evade or survive therapeutics designed to eliminate them. It also contributes to the notoriety of cancer to be incurable, leading to poor prognosis and patient mortality. Resistance towards chemotherapeutic agents complicates patient condition, as it may indicate malignancy. Reports have shown that over 90% of cancer-related deaths can be linked to chemoresistance [17]. The resistance to anticancer drugs can be classified into intrinsic or acquired resistance based on the time it is formed. Intrinsic resistance is identified as innate resistance and happens prior to drug administration. This phenomenon can be caused by inherited genetic mutations in tumors, the presence of insensitive subpopulations preexisting in heterogenous tumors, and activation of intrinsic pathways used as defense against external agents [9]. Meanwhile, acquired resistance can be categorized as the reduction of anticancer drug efficacy gradually after drug treatment, which can be caused by the activation of a second proto-oncogene that becomes the newly emerged driver gene. The mechanisms involved include mutations of drug targets and gradual changes in tumor microenvironment after treatment [9].

### 2.2. Mechanisms of Chemoresistance

Fundamentally, the chemo-resistant quality of tumors can be sourced from cancer stem cells (CSCs) and their ability to evade apoptosis. A small percentage of CSCs in heterogenous tumors can cause them to become chemo-resistant and subsequently malignant [18]. Treating resistant cancer cells may also be a challenge due to various chemoresistance mechanisms involved, some of which are drug efflux mechanisms, genetic and epigenetic mutations, oncogenic signaling, and tumor microenvironment interactions [18]. The most common chemoresistance mechanism is through drug efflux by ABC transporters that actively eject drug molecules from cancer cells. Some well-studied ABC drug transporters are P-glycoprotein (Pgp), multidrug-resistant protein 1 (MDR1), and ABCG2, also known as breast cancer-resistant protein [11]. The overexpression of ABC transporters is common in different types of chemo-resistant cancers, including leukemia, ovarian, and breast [19]. The expression of different drug transporters also contributes to resistance to different chemotherapeutic agents. For instance, overexpression of MDR1 transporters renders increased drug efflux such as doxorubicin, vinblastine, and digoxin [20], while ABCG2 overexpression is commonly observed in mitoxantrone-resistant breast cancer [20].

Nonetheless, drug efflux alone does not paint the complex picture of chemoresistance in cancer. Endogenous mechanisms, usually dictated by the genetic makeup of tumors, play a role as well. Genetic or epigenetic changes that render the drug ineffective are via avoiding or blocking the apoptosis pathway or overcoming proapoptotic signaling of the administered drug. A common driving mechanism in cancer is *TP53* mutation. In normal condition, the *TP53* gene is activated to halt cancer cell proliferation at cell cycle checkpoints. When DNA damage is detected, the cells will repair these damages, and when the damages are irreparable, p53 will initiate a series of signaling cascade that induces apoptosis to prevent the mutated cell from replicating. In cancer cells with *TP53* mutation, the apoptotic response is blocked; hence, they cannot replicate [10]. Up to 50% of all human cancers have a mutated *TP53* gene [10], which consequently increases resistance to drugs aimed at inducing apoptosis [21]. Genetic mutations such as that of p53 and Bcl2 are examples of chemoresistance arising from abnormal gene functions. Besides, chemoresistance caused by aberrant metabolic or enzymatic activity has also been recorded. For instance, 5-fluorouracil (5-FU), a common chemotherapeutic drug, is converted into several forms of uracil analogue, which disrupts RNA synthesis and inhibits thymidylate synthase from converting deoxyuridine monophosphate (dUMP) to deoxythymidine monophosphate (dTMP). This reaction provides a source of thymidylate for DNA replication and repair [22]. An overexpression of thymidylate synthase or other mechanisms that can salvage thymidylate can easily overcome the effects of 5-FU [22]. Similarly, abnormal enzyme activity such as glutathione S-transferase may degrade drug particles in cells [21].

The above-mentioned are just the common examples of the multitude of mechanisms involved in chemoresistance. Various studies have also been done to elucidate the involvement of other molecules (e.g., microRNAs (miRNAs), small-interfering RNAs (siRNAs)), tumor microenvironment, and drug activity in their roles and contribution to chemoresistance [17, 23–26]. More often than not, there are multiple resistance mechanisms acting against a single drug [21].

#### 2.2.1. Contribution of Epithelial-Mesenchymal Transition to Chemoresistance

Malignancy of a tumor is what defines its cancerous nature. The ability of tumors to grow and spread uncontrollably also contributes to the occurrence of chemoresistance by bestowing benign cells to a more resilient nature against chemotherapeutic drugs. Combining the effects of CSCs present in the tumor and the effects of malignancy, the process of metastasis causes a snowball effect in which drug resistance is more prominent, and the cells are harder to kill. Hanahan and Weinberg identified six hallmarks of cancer that characterizes malignant tumors [12], which enable increased survival of cancer cells [27]. One of them is the evasion of apoptosis to overcome the cytotoxic effects of drugs [12, 27]. Furthermore, cancer cells can metastasize and migrate, which is mediated by EMT [12]. EMT is also identified as a contributor to chemoresistance [28].

Biologically, EMT can be observed in embryonic development, tissue regeneration, and wound healing. In cancer, EMT is often discussed in the context of invasion and metastasis. During EMT, malignant features are induced in benign tumor cells, including stem-like characteristics, immune evasion, apoptosis inhibition, altered cell metabolism, and chemoresistance [13, 29–31]. Although the exact mechanisms are not yet clearly understood, EMT has been reported to play a crucial role in chemoresistance [28]. This claim may also be due to the fact that the occurrence of EMT is linked to CSC activity in the tumors [32]. During the transition process, cancer cells transform into mesenchymal-like cells by expressing mesenchymal markers, which is also accompanied by increasing CSCs with self-renewing abilities simultaneously, thus reducing their susceptibility to the cytotoxic effects of chemotherapeutic drugs [33, 34]. Furthermore, both chemoresistance and EMT share similar regulatory signaling pathways, including Notch, phosphoinositide 3-kinase/protein kinase B/glycogen synthase kinase-3 beta/Snail (PI3K/Akt/GSK-3*β*/Snail), and mitogen-activated protein kinase/c-Jun N-terminal kinase (MAPK/JNK) [28, 35]. Other evidences have also suggested that chemoresistance arising from quiescent CSCs shares an overexpression of EMT-inducing transcription factor zinc-finger E-box-binding homeobox-2 (ZEB2) [32, 36]. This finding further strengthens the implication that EMT in malignant tumors is strongly linked to CSC activity. Even more surprising, EMT is not only an activity commonly observed in CSCs, but it is also involved in the formation of CSC or cancer cells with stem-like properties [37–40].

However, targeting CSCs may not be ideal for many scenarios. CSCs replicate relatively slow to produce differentiated nonstem daughter cells that form the bulk of tumors. Most chemotherapeutic drugs target rapidly dividing cells, which make CSCs elusive to the effects of chemotherapy [39]. It is possible to suppress or abrogate CSCs by targeting prosurvival signaling pathways such as Notch, Wnt, epidermal growth factor receptor (EGFR), insulin-like growth factor (IGF), Akt, and PI3K. However, the signaling pathways often work simultaneously; thus, simultaneous inhibition is required for effective targeting [39]. Hence, targeting EMT process might seem to be the next ideal strategy. By reducing the mechanisms of cancer migration, malignant and chemo-resistant features might be reduced or inhibited [41, 42].

## 3. Matrix Metalloproteinases

**Figure 2:**
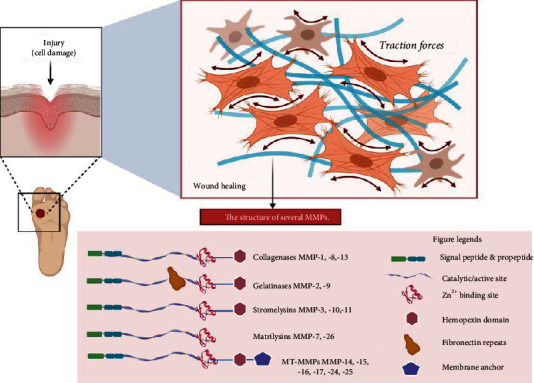
Structure of different types of MMPs. All MMPs are characterized by the chelated zinc in their structure, while each family can be distinguished based on other structural features such as fibronectin repeats in gelatinases and a membrane anchor in membrane type MMPs.

MMPs are a class of endopeptidases, which are known for the presence of chelated zinc and calcium in their structures. They are important in extracellular matrix (ECM) degradation. To date, 28 MMPs have been successfully identified in vertebrates, with 24 in humans, including two equivalent forms of MMP-23 (e.g., MM-23A and MMP-23B) that are encoded by two distinct genes on chromosome 1 [43]. The general structures of the MMPs classes in humans are depicted in Figure 2. Ten MMP genes are located on chromosome 11, whereas dissimilar chromosomes fix other MMPs. Generally, MMPs play a vital role in the activation and release of different chemokines, cytokines, growth factors, adhesion molecules, and cytoskeletal proteins, allowing them to contribute to physiological events like tissue repair, morphogenesis, inflammation, embryogenesis, wound healing, angiogenesis, and bone remolding [44]. Unlike in cancer tissues, MMP expressions are tightly regulated and found in low concentrations in normal tissues [45]. Dysregulation of MMP expression can cause diseases such as arthritis, ulcers, fibrosis, and cancer. In fact, MMP expression is raised in most cancer types and continuously accompanied by poor prognosis [46], as ECM degradation is a crucial step in EMT and cancer cell invasion and metastasis.

MMPs can be classified according to the substrate catalyzed, namely, collagenases, gelatinases, stromelysins, matrilysins, membrane-type MMPs (MT-MMPs), and other MMPs [47, 48]. Collagenases include MMP-1, MMP-8, and MMP-13. The main substrate cleaved by them are interstitial collagen (e.g., collagen types I, II, and III) [49], known ECM molecules, and other bioactive molecules such as interleukin 8 (IL-8), protease-activated receptor-1, and insulin-like growth factor-binding proteins [50], while gelatinase family consists of gelatinase A (MMP-2) and gelatinase B (MMP-9) in which they are identified by a region of fibronectin repeats in their structure. The fibronectin repeats enable the cleavage of large gelatinous substrates, including laminin, elastin, fibrillin, aggrecan, proteoglycans, and several ECM-like collagens (e.g., collagen types I, IV, V, VII, IX, X, and XI) [51, 52]. The gelatinase family is also commonly involved in ECM degradation and cell migration [53]. Stromelysins are similar to collagenases, but with the exception that they do not cleave interstitial collagen. Additionally, they also activate pro-MMPs by cleaving the propeptide domain in their structure. For instance, stromelysin-1 (MMP-3) cleaves pro-MMP-1 [52]. Next, matrilysins are identified by the lack of hemopexin domain, and they degrade ECM molecules such as laminin and type IV collagen [52]. As for MT-MMPs, they have an additional transmembrane domain or membrane anchor in their structure, and they commonly cleave collagens, laminin, fibronectin, and fibrin [49, 54].

### 3.1. Synthesis, Activation, and Inhibition of MMPs in a Biological System

MMPs are basically synthesized as proenzymes and become an active protease via proteolytic processing by removing the N-terminal inhibitory prodomain and exposing the catalytic site of the MMP enzyme. For MMP activation, it occurs both extracellularly and intracellularly [55–57]. Many MMPs (e.g., MMP-28, MMP-11, and MT-MMPs) in their propeptides demonstrate furin cleavage motif RXK/RR. In the trans-Golgi network, the furin-like proprotein convertases process MMPs. Moreover, proteolytic cleavage is required to inactivate the inhibitory prodomain. If furin alone releases the prodomain, the noncovalently associated intact prodomain results in the remaining inhibited MMP-14 (MT1-MMP) enzyme [58]. Serine proteases such as plasmin, MT-MMPs (e.g., MMP-14 activated MMP-2 proenzyme), or other active MMPs (e.g., MMP-3 activated proenzymes of MMP-1 and -9) mediate the activation of soluble MMPs. Due to the overlapping cleavage preferences, MMPs have functional redundancy. Consequently, studies have shown that MMP knockouts in mice are nonlethal and do not demonstrate a strong phenotype. However, MMP-14 knockout mice develop bone malformations, dwarfism, and die before adulthood, supporting the role of MMP-14 in both collagen turnover and cell migration during gastrulation [58–62]. Interestingly, mice that lack both MMP-14 and MMP-2 die immediately after birth [63]. Once MMPs are activated, tissue inhibitors of metalloproteinases (TIMPs) are induced to inhibit MMPs. TIMPs in humans have four different types (e.g., TIMP-1, -2, -3, and -4) [64, 65]. The balance of MMP/TIMP is an important factor in regulating the net proteolytic activity of MMPs.

### 3.2. Interactions between EMT and MMPs

As described previously, EMT-mediated chemoresistance can be attributed to MMP activity. Upon activation of EMT, cancer epithelial cells that display apical-basal polarity and cell-to-cell adherence by E-cadherin slowly lose their structural integrity and morphology. Gradually, E-cadherin expression is suppressed and replaced by the expression of mesenchymal markers such as N-cadherin, vimentin, and fibronectin [66]. EMT is induced by several transcription factors, including Snail, ZEB1, and ZEB2 and beta helix-loop-helix proteins [67, 68]. Among the effects of these transcription factors, increased expression of proteases (e.g., MMP-2 and MMP-9) are what cause cancer cells to undergo EMT for promoting cellular detachment and invasiveness [68]. During EMT, the serum protease plasmin cleaves the inactive MMP proproteins to activate them, which then degrade basal membrane and ECM components to allow metastatic cells to migrate [69]. The cleavage of E-cadherin is attributed to MMP activity, which converts E-cadherin to soluble E-cadherin (sECAD) to induce paracrine signaling of EMT via EGFR [70]. It has been known that decreased E-cadherin expression is accompanied by increased N-cadherin expression, also reflecting the increased degradation by MMPs, particularly MMP-9, -10, and -15 [71, 72].

Other metastatic growth factors are also released during MMP activity via ECM degradation to possibly promote angiogenesis, which is another important step in cancer metastasis [73]. One example is that MMP-9 enhances TGF-*β* activity, which promotes cancer invasion in metastatic cancers [52, 74]. Besides, MMP-9 also can reduce IL-2 response, which may contribute to immune evasion by cancer cells [75]. Several inflammatory mediators in the tumor microenvironment have been implied to possess tumor-promoting effects that enhance survivability, proliferation, and chemoresistance [76]. MMP activity also modulates anti- and proinflammatory effects, which might explain the resistant nature of advanced tumors [52].

### 3.3. Diagnostic and Prognostic Values of MMPs

Detection levels of molecules from resected tumors can provide a multitude of information to formulate a treatment strategy based on the patient's condition. Due to the roles of MMPs in cancer metastasis, several studies have linked their expression and corresponding effects on cancer prognosis and patient survivability. MMP expression has been implied to provide a valuable indication for diagnosis and prognosis. Generally, an elevated level of MMP expressed often signifies a poorer prognosis and a lower chance of survivability [77]. For example, elevated levels of MMP-1 are indicative of advanced breast cancer and signifies poor prognosis [78, 79]. This feature is also common, particularly with the overexpression of gelatinases [80, 81]. Investigations on glioma, gastric carcinoma, and advanced laryngeal carcinoma have proven that gelatinases are a huge contributor to poor prognosis in cancer patients [81–83]. Further finding also revealed that MMP-2 and MMP-9 may be responsible for glioma recurrence and malignancy [82].

Several stages in tumor development may require different sets of effectors throughout the progression. Different MMPs have been demonstrated to be correlated to different stages of cancer development. For instance, Juchniewicz reported that mRNAs of MMP-7, MMP-10, TIMP-1, and TIMP-2 were overexpressed in patients with esophageal cancer [84]. They further found that MMP-10, TIMP-1, and TIMP-2 were correlated to tumor size, with TIMP-2 had the most significant impact on tumor size. Besides, MMP-7 also correlated to disease stage and progression as well as lymph node metastasis [84]. Another study suggested that differential expression of MMP-9 could contribute to breast cancer heterogeneity to identify different subtypes. This study also linked MMP-9 overexpression to high-grade breast cancers such as triple-negative breast cancer and HER2-positive breast cancer subtypes, which are also related to relapses and lymph node metastases [85].

Although the sole expression of a single MMP is valuable on its own, however, molecular signatures involving two or more MMPs expression can provide more precise information to be used as a diagnostic or prognostic tool. Some MMPs cleave pro-MMPs to activate other MMPs. For instance, MMP-14 cleaves pro-MMP-2, thus making sense that dysregulation of one MMP may affect the expression of another MMP [86]. Therefore, MMP combinations can be used as diagnostic biomarkers. Gobin demonstrated that the combined expression of MMP-11 and MMP-19 signified a strong correlation to thyroid cancer, which is stronger than individual MMPs [86]. Thus, identifying unique signatures of *MMP* genes may be useful to develop pan-cancer biomarkers. Furthermore, samples can be obtained in a less invasive manner by collecting from patients' circulatory system as compared to extracting biopsies directly.

Apart from the above, it has been shown that synergism between MMPs and other molecules also has prognostic potential. For instance, Wang et al. observed that a low expression level of MMP-9 with high expression of TIMP-2 provided the best overall survival prognosis for colorectal cancer patients [87]. This study showed that interacting molecules (e.g., TIMP-2) regulating MMP activity were also valuable for their use in diagnosis and prognosis. TIMP-2 is known to bind favorably to MMP-9 among other MMPs. The tissue inhibitor complexes with the catalytic zinc cofactor inactivate MMPs [88], which may explain the synergistic effect of TIMP-2 and MMP-9 as compared to both with high or low expression [87]. Another finding by Ren et al. showed a significant impact on the survival of patients with gastric cancer based on the positive expression of COX-2 and MMP-13. Due to the fact that both proteins are highly involved in gastric cancer progression and tumor invasion, thus, they proposed that COX-2 and MMP-13 expression can be used as a reference index for treatment strategy and a mean of disease prognosis [89].

Despite the promising prospect of utilizing MMPs as diagnostic and prognostic tools, care should be taken when analyzing MMP expression to determine patient prognosis. As described above, not all cancer types reflect similar findings with similar expression profile; thus, MMP expression can mean differently across different cancers. Hence, extensive studies are required to further confirm the utilization of MMPs as a cancer biomarker in different cancers.

### 3.4. Biological and Regulatory Roles of MMPs in Chemoresistance

**Figure 3:**
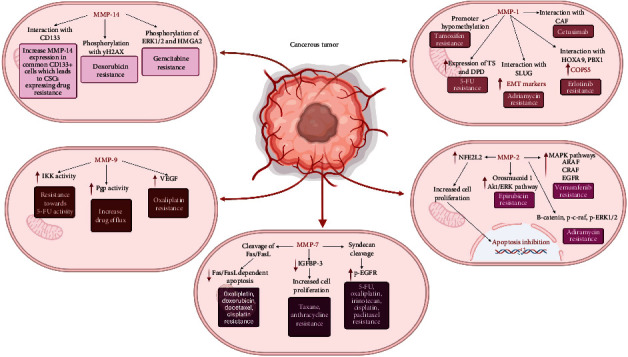
Associated mechanisms of MMPs and their effect on chemoresistance. MMP activity generally contributes to chemoresistance via EMT induction and apoptosis resistance, both of which increase cell survivability and overcome chemotherapeutic drug effects. However, several MMPs can cause chemoresistance via other pathways such as increase in pathways such as Akt, EGFR, and MAPK pathways. Other specific mechanisms such as Fas cleavage and inhibition of cell cycle arrest also contribute to chemoresistance.

Although various MMPs play a role in cancer phenotypes, particularly cell migration and proliferation, several MMPs have been identified to be more critical in promoting metastasis and hence chemoresistance. This section discusses the biological roles of selective MMPs that are implicated in chemoresistance and associated molecular mechanisms (Figure 3) as well as their regulatory roles or interaction with molecules related to this phenomenon, with the relevant information for selective MMPs are summarized in Table 1.

#### 3.4.1. MMP-1

MMP-1 belongs to the collagenase family of MMPs, and it cleaves several substrates such as fibrillar and nonfibrillar collagen types I, II, III, VI, VII, VIII, and X, laminin, casein, serpin, and MMPs -1, -2, and -9 [52]. Elevated MMP-1 activity has been reported to contribute to poor prognosis outcomes in breast cancers [78, 79]. It is also responsible for events during EMT that facilitate cancer cell migration and invasion. The movement of cancer cells is facilitated by channels formed in the ECM that is regulated by MMP-1 activity [78]. Aside from its role in ECM degradation, MMP-1 can activate latent molecules to promote downstream prooncogenic signaling pathways-related proteins such as VEGF, EGF, and CXCL-12, which consequently activate metabolic-related transcription factors and pathways involved in cancer, including hypoxia-inducible factor-1 (HIF-1), MAPK, and extracellular-signal-regulated kinase (ERK) pathways [90, 91]. Furthermore, MMP-1 also activates other MMPs that are implicated in metastasis [52, 90].

In a study carried by Kim et al. [92], it was found that MMP-1 upregulation rendered MCF-7 breast cancer cells to be resistant towards tamoxifen treatment [92]. The study further revealed that MMP-1 expression was upregulated by hypomethylation in both tamoxifen-resistant breast cancer tissues and MCF-7 cells. Besides, short hairpin RNA (shRNA-)-mediated MMP-1 gene silencing restored the sensitivity of MCF-7 cells towards tamoxifen, as evident in apoptosis induction [92]. These findings suggest that MMP-1 promoter hypomethylation could be the mechanism of driving tamoxifen resistance; however, further investigations are required.

Another study also identified that MMP-1 is a player in causing 5-FU resistance in nasopharyngeal carcinoma cells [93]. It was observed that *MMP-1* gene silencing inhibited cancer cell growth and promoted apoptosis. Comparatively, *MMP-1* gene silencing showed higher inhibitory rate in cell proliferation and invasion than 2.5 mg/mL of 5-FU treatment alone. Interestingly, the chemosensitivity of cancer cells was further provoked via the downregulation of thymidylate synthase and dihydropyrimidine dehydrogenase, which are two crucial enzymes involved in 5-FU metabolism [93].

Moreover, it was reported that resistance towards cetuximab induced by cancer-associated fibroblasts (CAF-)-induced cetuximab resistance in head and neck squamous cell carcinoma was mediated by MMP-1 expression [94]. In the study, mRNA and protein levels of MMP-1 were overexpressed in UT-SCC-9 cells cocultured with CAF. When MMP expression was downregulated using MMP-1 inhibitor and in siRNA-treated CAF, cancer cells were partially protected from the cytotoxic effect of cetuximab treatment. These findings indicate that MMP-1 may protect UT-SCC-9 cells from cetuximab treatment together with other MMPs. Furthermore, inhibition of MMP-1 expression using siRNA significantly reduced the protective effect against cetuximab [94]. However, exact mechanisms and pathways of the resistance mediated by MMP-1 were not yet elucidated in this study. Similarly, another study also reported Taxotere resistance in breast cancer cells cocultured with CAF [95]. The inhibition of MMP-1 expression by GM6001, an inhibitor of MMP-1, increased chemosensitivity towards Taxotere treatment, which was evident from decreased cell proliferation and invasion assays and increased apoptotic activity [95].

Furthermore, several studies have identified MMP-1 overexpression to be a contributing factor of multidrug resistance [96, 97]. MMP-1 was reported to be upregulated in Adriamycin MCF-7 breast cancer cells, along with MMP-2 and MMP-9 [97]. It was also speculated that slug acts as a promoter for MMP-1 overexpression, leading to increased levels of a EMT marker (i.e., N-cadherin) and decreased levels of an epithelial cell marker (i.e., E-cadherin). Further investigation with siRNA-mediated *MMP-1* gene silencing in breast cancer cells showed an increase in apoptosis by Adriamycin, suggesting that MMP-1 plays a crucial role in the chemoresistance of breast cancer cells towards Adriamycin treatment [96].

Interestingly, MMP-1 overexpression also confers to erlotinib resistance in nonsmall cell lung cancer [98]. The study used in silico methods to screen for gene profiling of differentially expressed genes from microarray data against Kyoto Encyclopedia of Genes and Genomes in human nonsmall cell lung cancer tissues and normal lung tissues. It was further discovered that COP9 signalosome subunit 5 (COPS5) was also overexpressed along with MMP-1 overexpression. A combination of MMP-1 and COPS5 overexpression conferred a poor overall survival; thus, COPS5 involved in erlotinib resistance mediated by MMP-1 overexpression. To validate the role of MMP-1 in erlotinib resistance, several transcription factors regulating MMP-1 expression were identified, among which HOXA9 and PBX1 were highly expressed. They have been previously reported to be associated with drug resistance [98–100]. Hence, several proposed mechanisms of erlotinib resistance mediated by HOXA9- and PBX1-induced MMP-1 upregulation include alterations of cell growth, apoptosis, protein phosphorylation, and angiogenesis [98].

In another study, MMP-1 induced perineural invasion in pancreatic cancer via MMP-1/protease-activated receptor-1/substance P/neurokinin 1 receptor (MMP1/PAR1/SP/NK1R) paracrine loop, which was activated by Akt [101]. Akt is known to regulate apoptosis and cell viability; thus, MMP-1 may target Akt pathway to modulate chemoresistance through cell survival promotion and apoptosis inhibition [99, 100]. It has been reported that Akt activation is related to resistance against apoptosis induced by TNF-related apoptosis-inducing ligand (TRAIL/APO-2L). Furthermore, Akt signaling phosphorylates cyclic AMP response element-binding protein (CREB) and I*κ*B-kinase (IKK) to regulate the expression of antiapoptotic genes [102]. Due to the fact that most survival signals are mediated by PI3K/Akt, thus, MMP-1 may alter cell response to apoptosis induced by antineoplastic drugs. However, additional molecular studies are required to further elucidate other possible pathways activated by MMP-1 in regulating chemoresistance and cancer metastasis.

#### 3.4.2. MMP-2

MMP-2 is also known as gelatinase A, and it is known to break down various substrates such as interstitial type I collagen and native type IV collagen, as well as more than 30 other substrates involved in ECM degradation. It has been discovered that MMP-2 overexpression across almost all types of cancer is linked to a feature of cancer aggressiveness and malignancy [102].

Although MMP-2 has been implicated in promoting chemoresistance [103], not much study has successfully described its exact mechanisms. The possible mechanisms generally include upregulation of EMT markers, which eventually lead to drug resistance. For instance, elevated Notch 1 signaling pathway was followed by the increased expression of MMP-2, Snail, and vimentin, leading to EMT-induced chemoresistance. The increased expression of EMT markers also contributed to apoptotic inhibition enhanced cell proliferation, as evident with a higher cell number in S and G2 phases. Besides, Rajesh et al. identified that transcription factor nuclear factor, erythroid 2-like 2 (NFE2L2) promoted temozolomide-induced chemoresistance in glioblastoma multiforme (GBM) cells by transcriptionally regulating MMP-2 [104]. It was also noted that the combined treatment of NFE2L2 inhibitor (diosgenin) and temozolomide rendered higher apoptotic cell number and increased early cell cycle arrest, along with reduced expression levels of MMP-2 [104]. Thus, it may be possible that MMP-2 induced chemoresistance by inhibiting apoptosis and increasing cell proliferation, which may have overcome the effects of chemotherapeutic drugs.

Qiong and Yin [105] revealed that epirubicin resistance in breast cancer was promoted by an elevated level of orosmucoid 1, which was induced by MMP-2 and MMP-9 upregulation. They also showed that Akt and ERK signaling pathways were also upregulated in causing the chemoresistance in breast cancer. It has been widely reported that both Akt and ERK signalings are largely associated with cell survival and apoptosis regulation. Thus, this study found that orosmucoid 1 upregulation in breast cancer cells increased MMP-2 and MMP-9 expression, activated Akt/ERK signaling pathways, all of which stimulating epirubicin resistance via apoptosis inhibition [105].

Furthermore, vemurafenib resistance in melanoma cells was attributed to the activity of MMP-2. In a study conducted on vemurafenib-naïve cells (SK-MEL-28N) and vemurafenib-resistant (SK-MEL-28R) cells, MMP-2 activity was observed to be consistently higher (>14-fold) in vemurafenib-resistant cells as compared to its nonresistant counterpart. It was discovered that MMP-2-mediated vemurafenib resistance involved MAPK pathway reactivation [106]. Upon vemurafenib treatment, SK-MEL-28N showed decreased MEK and ERK phosphorylation, while SK-MEL-28R cells expressed ARAF reactivation and BRAF inactivation. SK-MEL-28R also showed CRAF reactivation and EGFR activation, all of which contribute to vemurafenib resistance [107].

In addition, MMP-2 has been reported to be highly expressed in several drug-resistant tumors [108, 109]. For instance, paclitaxel-resistant HeLa and ME180 cervical cancer cells showed increased MMP-2 activity, along with other EMT markers such as *β*-catenin and p-c-raf [108]. Adriamycin resistance in osteosarcoma was also associated with an elevated MMP-2 activity. In the study, Ren et al. [109] established a Adriamycin-resistant cell line, ADM-MG-63. ADM-MG-63 cells showed increased MMP-2 and p-ERK1/2 protein levels, as evident in Western blot analysis. Conclusively, this study indicates that MMP-2 plays a role in Adriamycin resistance in melanoma mediated by p-ERK1/2 and thus targeting MMP-2/ERK1/2 may be a new targeted therapy for melanoma patients [109].

#### 3.4.3. MMP-7

Matrilysin-1 or MMP-7 is the smallest member of the MMP family as it lacks the hemopexin domain, which is found in almost all other MMPs [110]. The substrates cleaved by MMP-7 are collagens, proteoglycans, laminin, and fibronectin [111]. Among the metastatic roles played by MMP-7, degradation of E-cadherin is perhaps one of the most crucial and well-studied proteolytic activities [106, 112, 113]. Cleavage of transmembrane E-cadherin by MMP-7 produces sECAD [70, 112]. sECAD induces the expression of MMP-2, MMP-9, and MMP-14 at the mRNA and protein levels, which consequently increases the proteolysis of the basement membrane. Furthermore, this biological proteolysis causes cancer cells to transform to more invasive mesenchymal phenotype. Similar to MMP-2, MMP-7 is also capable of cleaving integrin, which reduces cell adhesion and enhances metastasis [110]. Integrins are also important in other tumor development processes such as cell proliferation, apoptosis, angiogenesis, and leukocyte migration [114]. Additionally, MMP-7 also activates pro-MMP-2, thus starting an enzyme cascade and increasing invasive activity [115].

Several mechanisms relating to chemoresistance have been discovered within the context of matrilysin activity. MMP-7 is well-known to interact with Fas/FasL system. Fas is a membrane protein of the TNF superfamily that binds to its natural ligand, FasL. The binding of FasL to Fas triggers a caspase-dependent apoptosis [116]. Studies have shown that MMP-7 can protect cancer cells from chemotherapeutic drugs by modulating Fas expression and activation as well as the cleavage of both Fas and FasL, thus blocking the Fas-dependent apoptotic effect of the drug [113]. It may also play a role in early tumorigenesis by selecting cancer cells with reduced sensitivity to Fas-induced apoptosis. In this manner, surviving cancer cells are inherently resistant to drugs (e.g., oxaliplatin) that trigger apoptosis via the Fas pathway [117]. Other evidence regarding elevated MMP-7 levels in drug-resistant cancers have shown the involvement of MMP-7 and Fas interaction in the acquisition of resistance towards chemotherapeutic drugs. For instance, doxorubicin resistance was induced by MMP-7 activity from which doxorubicin-induced apoptosis was inhibited by soluble Fas cleaved by MMP-7 [118]. Other than that, it was found that elevated levels of MMP-7 and soluble Fas in serum of prostate cancer patients also conferred to docetaxel resistance [119]. In addition to doxorubicin and docetaxel, increased MMP-7 mRNA expression was also found to be associated with cisplatin resistance in head and neck cancer cells, as evident in Gene Ontology analysis [120].

IGFBP-3 was also found to be a target of MMP-7 degradation to cause drug resistance after chemotherapy [121]. It is responsible for binding IGF-I to regulate the binding of IGFs to their receptors in modulating cell proliferation and differentiation [122]. Gallego et al. found that MMP-7 levels were elevated after chemotherapy, degraded IGFBP-3, and induced acquired chemoresistance against anthracycline and taxane [121]. Although the exact mechanisms underlying increased MMP-7 expression postchemotherapy are not fully understood, hypoxic conditions are suggestive to play a role in MMP-7 expression, due to the fact that *MMP-7* gene expression is highly induced under hypoxic conditions [123]. It is plausible that MMP-7 degraded IGFBP-3 in hypoxic condition to reduce cancer cell apoptosis. In another study, Liu et al. discovered that MMP-7 exposure in lung adenocarcinoma cells increased Bcl-2 expression to inhibit cell death [124]. The study further identified the possible pathways in cisplatin chemoresistance, of which the mitochondria-mediated pathway of apoptosis was inhibited by increased Bcl-2 protein levels, thus inactivating proapoptotic Bax proteins [117, 119].

MMP-7 was also reported to induce chemoresistance via the shedding of syndecan-1 (Sdc-1). Wang et al. [127] recruited colorectal patients treated with chemotherapeutic agents (i.e., 5-FU, oxaliplatin, irintecan, cisplatin, or paclitaxel), and Sdc-1 serum levels were compared in whereby preoperative and postoperative patients with healthy controls. The measurement of Sdc-1 serum levels found that it was higher in preoperative patients followed by postoperative and healthy controls. It was also noted that preoperative patients with high Sdc-1 serum levels were less responsive to five chemotherapeutic drugs l as compared to postoperative patients, which was supported by a poorer disease-free survival rate. Sdc-1 serum levels and MMP-7 levels were positively correlated. The associated molecular mechanism of chemoresistance in colorectal patients was caused by MMP-7-mediated Sdc-1 shedding that consequently promoted EGFR phosphorylation and downstream signalings [125].

#### 3.4.4. MMP-9

MMP-9 is another member of the gelatinase family and has been observed to cleave a multitude of different substrates, including nonfibrillar collagens, gelatin, elastin, and fibrillin [52, 126]. It is by far the most well-studied MMP in regard to cancer metastasis and tumor progression. This is due to its ubiquity in various cancer types and its role in EMT and cancer invasion as a whole. As previously mentioned, the gelatinase family is the most commonly observed to indicate poor prognosis in cancer patients.

ECM degradation during metastasis is regulated by the activity of gelatinases, where MMP-9 promotes invasion by hydrolyzing the physical barrier of the ECM comprising gelatins and type IV, V, XI, and XVI collagens. Following the degradation of the basement membrane, cancer cells migrate into the bloodstream or the lymphatic vessel [52]. The role of MMP-9 in inducing EMT is predominantly promoting cancer metastasis. For instance, Li et al. demonstrated that EMT could not be proceeded without MMP-9 activity. The study induced EMT in thyroid cancer cells using oncogenic factor TGF-*β*1. However, *MMP-9* gene silencing in thyroid cancer cells using shRNA constructs inhibited TGF-*β*1-mediated EMT. The results further showed that EMT was halted, as evident by the increased expression of E-cadherin and decreased expression of vimentin. The significance of this study implied that MMP-9 could act as a key driver of EMT. Hence, EMT-induced chemoresistance is directly associated with MMP-9 overexpression. Due to this aspect, many studies have been conducted to identify target molecules that induce MMP-9 expression and inhibit EMT-mediated chemoresistance by suppressing MMP-9 [127–130].

Signal transducer and activator of transcription 3 (STAT3) signaling pathway was reported to induce chemoresistance and EMT via MMP-9 induction in bladder cancer [131]. NF-*κ*B was implied to contribute to metastasis and chemoresistance in colorectal cancer via MMP-9 upregulation, causing resistance to 5-FU [127]. The proposed mechanism was attributed to the oscillatory activation of NF-*κ*B that induced tumor necrosis factor-alpha (TNF-*α*)-dependent *MMP-9* gene expression and chemoresistance arising from 5-FU-stimulating NF-*κ*B to induce IKK activity.

Laios et al. successfully enhanced chemosensitivity in ovarian cancer cells using a selective MMP-2/MMP-9 inhibitor, which showed higher MMP-9 specificity at lower concentrations. The inhibitor used also induced proapoptotic function when combined with TNF-*α*, TRAIL, or FasLs, which may indicate the possible mechanisms by which MMP-9 induced chemoresistance [132]. Another study by Asuthkar et al. suggested that increased drug resistance in glioma cells was likely caused by increased Pgp activity in glioma CSCs [128]. The expression levels of Pgp and multidrug resistance protein (MRP) were increased after chemotherapy in GBM patients. The effects of miR-211 and MMP-9 suppression on chemosensitivity were evaluated by performing the rhodamine 123 efflux assay to measure Pgp-mediated efflux, as evident by decreased rhodamine 123 intracellularly. The findings showed that a reduction in Pgp efflux in glioma cells could be achieved by suppressing the expression of miR-211 and/or MMP-9 [128]. Furthermore, MMP-9 overexpression may lead to chemoresistance by upregulating VEGF [133]. The expression of MMP-9 is mostly accompanied by decreased E-cadherin expression and increased VEGF expression. Based on the study conducted via collagen gel droplet–embedded culture–drug sensitivity test (CD-DST), it was indicated that the positive expression of VEGF and negative expression of E-cadherin were associated with oxaliplatin resistance [137]. However, the exact mechanisms of oxaliplatin resistance were not clearly elucidated.

#### 3.4.5. MMP-14

Unlike other MMPs discussed previously that are secreted MMPs, MMP-14, commonly known as MT1-MMP, is a membrane-bound proteinase. There are six MT-MMPs bound to the cell surface by a COOH-terminal transmembrane domain or a glycosyl phosphatidyl anchor [134]. The substrates cleaved by MMP-14 include gelatin, collagens, fibronectin, laminin, aggrecan, and perlecan. Other substrates cleaved by MMP-14 include cell surface molecules such as mucin 1, tTG, integrins, and CD44 molecule [52, 110]. Apart from degrading ECM components, MMP-14 is also responsible for the activation of pro-MMP-2, pro-MMP-9, and pro-MMP-13, of which pro-MMP-2 and pro-MMP-9 activation have been claimed as a crucial step in cancer cell invasion and metastasis [135, 136].

A study investigating the invasive potential of CD133^+^ endometrial cancer cells found that CD133^+^ cells had CSC potential and showed resistance towards chemotherapeutic drugs such as cisplatin and paclitaxel. The study inferred that the mechanism behind drug resistance in CD133^+^ cells was due to the elevated MMP-14 expression as compared to other MMPs [137]. *MMP-14* silencing analysis revealed that MMP-14 activity is crucial in determining the invasiveness in both CD133^+^ and CD133^−^ cells. This study possibly implied that MMP-14 regulated CSCs to implicate in cancer invasion and chemoresistance. The findings in this study are also mirrored in another investigation on the role of MMP-14 in brain CSCs [138], whereby MMP-14 dictates the regulation and formation of CD133^+^ cells and brain CSCs, which are contribute to chemoresistance. The study suggested that targeting MMP-14 could reduce EMT induction and chemoresistance by inhibiting the formation of brain CSCs [32, 138].

MMP-14 was also implied to contribute to poor response to chemotherapy in triple-negative breast cancer [65]. The analysis using Kaplan-Meier plot revealed that MMP-14 expression in triple-negative breast cancer tissues was inversely correlated to therapeutical response when measured in relapse-free survival and overall survival. Furthermore, the inhibition of MMP-14 expression in MDA-MB-231 cells could sensitized them to the combination treatment of ionizing radiation and doxorubicin. The proposed mechanism of chemoresistance by MMP-14 was suggested to be via affecting DNA double-strand breaks. The inhibition of MMP-14 expression increased the phosphorylation of DNA damage marker, *γ*H2AX [65]. Hence, it is possible that MMP-14 overexpression protects cell apoptosis by reducing DNA damage caused by radiotherapy and chemotherapy.

In another study, MMP-14 was found to play a mediation role in gemcitabine resistance in in pancreatic cancer cells [139]. It was found that the collagen proposed mechanism of gemcitabine resistance is via the phosphorylation of ERK1/2 and increased expression of high mobility group A2 (HMGA2) by MMP-14 activity. Because HMGA2 was shown to protect pancreatic cancer cells from DNA damage-induced apoptosis, elevating MMP-14 expression will consequently reduce the effects of gemcitabine by increasing the expression of MMP-14. Furthermore, *MMP-14* gene silencing using siRNA also reduced pERK1/2 and HMGA2 [139]. Although further research still needs to be done, MMP-14 largely contributes to chemoresistance via cell cycle arrest inhibition.

## 4. Targeting MMPs to Enhance Chemosensitivity in Cancer Therapy

Due to the well-known roles of MMPs in cancer progression and EMT, which consequently lead to chemoresistance, thus, inhibition of MMP activity in the extracellular space has been extensively studied as an approach for adjunct cancer treatment to traditional cytotoxic drugs. This action may sensitize cancer cells towards the effects of chemotherapeutic agents. The earliest MMP inhibitors (MMPIs) tested for therapy are peptidomimetics designed to mimic the natural ligand of MMPs such as collagens. Batimastat, the first MMPI to be tested in clinical trials, is a hydroxamic acid moiety that chelates the zinc in MMPs [140]. Many more MMPIs soon follow in development. Despite the fact that MMPIs have shown good efficiency against malignant tumors in preclinical settings, the outcome in clinical trials have been disappointing thus far, largely due to their broad-spectrum affinity, causing various side effects. Thus, it is a prerequisite to explore and identify the promising MMPI for targeting MMPs in adjunct cancer therapy, particularly in enhancing the chemosensitivity of cancer cells.

### 4.1. Current and Potential Therapeutics Targeting MMPs in Chemoresistance

As discussed, MMP levels are typically low in normal conditions and overexpressed in pathological conditions. Interestingly, the elevated expression levels of MMPs are in line with cancer severity [144]. As such, the interest in inhibiting MMP expression and activity has gained attention as a possible drug target to reduce chemoresistance. Over the decades, various MMPIs have been developed to target and inhibit MMPs expression and activity. However, drug resistance and toxicity associated with it have become a hindrance for their clinical application [142, 143]. The information of current and potential MMPIs and their preclinical and/or clinical trial status are summarized in Table 2.

**Figure 4:**
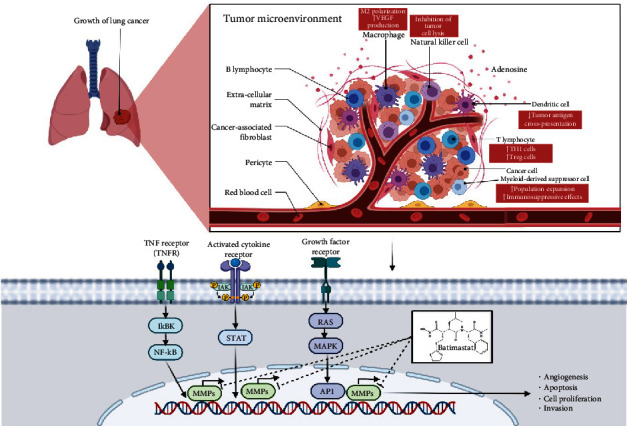
The mechanism of action of Batimastat in cancer therapy targeting MMPs. It is suggested that inhibiting MMPs can improve chemosensitivity and reduce cancer prognosis. Abbreviation: IkBK: inhibitory-*κ*B Kinase; NF-*κ*B: nuclear factor-kB; JAK: Janus kinase; STAT: signal transducer and activator of transcription; RAS: rat sarcoma virus; MAPK: mitogen-activated protein kinase; AP1: activator protein 1.

One of the earliest strategies to target MMPs is by chelating the zinc ions in their structure. Small molecules such as hydroxylamine are known to chelate Zn^2+^ and became the first prototype of MMPIs to enter drug trials [144]. Examples of hydroxamic acid-based inhibitors include batimastat and marimastat. Early studies showed that this can retard tumor growth [145]. Furthermore, batimastat was also shown to possess chemosentizing effects in GBM cells towards temozolomide (Figure 4) [146], while prinomastat (AG3340) sensitized nonsmall cell lung cancer towards carboplatin [147]. However, clinical trials soon proved that the side effects of these MMP inhibitors outweigh the benefits. The most common side effect associated with hydroxamic acid inhibitors is musculoskeletal syndrome, which is likely caused by simultaneous targeting multiple MMPs and possibly other non-MMP enzymes [144]. Following these, research on MMPIs has shifted towards other approaches such as inhibitory antibodies against MMPs [144]. Several antibody inhibitors have been developed, such as andecaliximab (GS-5745) and DX-2400, which have shown promising results in early clinical and preclinical trials, respectively [148, 149]. However, no study has been done whether both inhibitors have chemosensitizing effects.

Studies on synthetic MMPIs have also been done, with promising results in *in vitro* experiments. One such study was conducted by Laios et al. using a selective MMP-9/MMP-2 inhibitor ((2*R*)-2-[(4-Biphenylsulfonyl) amino]-3 phenylpropionic acid (C21H19NO4S)) [132]. The study concluded that treatment of cisplatin-resistant A2780cis ovarian cancer cells with C21H19NO4S and cisplatin enhanced cisplatin-induced cell death, indicating a chemosensitizing effect. The study also tested the effectiveness of different treatment modes, and it was concluded that pretreatment of C21H19NO4S followed by cisplatin had a greater chemosensitizing effect compared to cotreatment [132]. However, the specific mechanism of action inhibiting chemoresistance in the cells is not yet fully elucidated in the study.

Other non-anticancer drugs may also possess chemosensitizing effects when combined with anticancer drugs. For example, metformin, a drug used to treat type 2 diabetes, was shown to inhibit MMP-9 expression [150]. When combined with sorafenib, it was able to induce chemosensitivity towards sorafenib in hepatocellular carcinoma cells. The proposed mechanism, which was investigated via Western blot analysis, showed that metformin inhibited phosphorylation of ERK1/2 and JNK1/2, thus inhibiting protein expression of MMP-9 and urokinase-type plasminogen activator (uPA). The chemosensitizing effect was reflected in the reduction of cell migration and invasion assays when the cells are treated with a combination of metformin and sorafenib, as compared to single-drug treatment [150].

### 4.2. Downregulation or Inhibition of MMPs to Sensitize Cancer Cells to Anticancer Drugs

Given that direct targeting of MMPs is met with limitations and challenges, inhibition or downregulation of activation pathways of MMPs may be an alternative strategy to treating metastasis and chemoresistance. Because activation pathways of MMPs also activate other metastatic signaling molecules, thus inhibiting or downregulating the expression of MMPs may cause biological effects other than chemosensitivity to be induced.

Yang et al. demonstrated that enalapril, a common antihypertensive drug, could induce chemosensitivity in colorectal cancer cells towards 5-FU [159]. The cotreatment of both drugs inhibited the NF-*κ*B/STAT3 pathway, which is responsible for activating several proteins, including MMP-2 and MMP-9. Enalapril alone did not significantly exhibit antiproliferative properties on colorectal SW620 and HCT116 cells, while 5-FU reduced the viability of SW620 and HCT116 cells by 20% and 51%, respectively, at 10 *μ*M. The reduction in cell viability was significantly increased to 83% in SW620 cells and 87% in HCT116 cells. Similar results were also observed in the liver metastasis model in nude mice injected with SW620 cells. Western blot analysis showed that the cotreatment of both drugs completely suppressed p-STAT3, p-p65, Cyclin D1, c-Myc, Bcl-2, X-linked inhibitor of apoptosis (XIAP), MMP-2, and MMP-9 as compared to the slight inhibition caused by 5-FU alone [159].

Another study also discussed the role of ETS1, a member of the ETS transcription factor family, in the induction of chemoresistance and invasion in paclitaxel-resistant prostate PC3 cancer cells [160]. It was interesting to note that ETS1 silencing using two types of siRNA could reduce the mRNA levels of MDR1 and MMP-9. Besides, MDR1 protein was reduced by 0.66-fold by siRNA1 and 0.46-fold by siRNA2, whereas MMP-9 secretion was abolished after silencing. These findings suggest that ETS1 overexpression promotes paclitaxel resistance by upregulation of MMP-9 and MDR1 [160]. Thus, inhibiting ETS1 expression may reverse paclitaxel resistance indirectly by downregulating MDR1 and MMP-9 expression.

Furthermore, a study by Wu et al. identified annexin A5 protein as a promoting agent of chemoresistance in GBM cells against temozolomide [161]. As annexin A5 was shown to be overexpressed in human GBM cells (e.g., U-87 MG and U-118 MG), the expression of annexin A5 was silenced by shRNA, which showed a significant reduction in cell invasion capability, MMP-2 expression, temozolomide resistance, and Akt phosphorylation in annexin A5-shRNA-treated cells. In the context of chemoresistance, up to 3-fold and 2-fold of the maximal inhibitory concentration (IC_50_) of temozolomide was required to inhibit annexin A5 expressing U-87 MG and U-118 MG cells, respectively, as compared to normal control cells. Upon treatment with A5-shRNA, the required concentration to inhibit the cells was only half of the IC_50_ obtained from normal control cells [166]. Hence, similarly inhibiting or suppressing annexin A5 potentiates chemosensitivity in GBM by downregulating MMP-2 expression.

Phytochemical composition such as alkaloids and phenolic compounds found in plants have shown to possess beneficial effects for treating various diseases, including cancer [162]. Miranda et al. [163] demonstrated that cernumidine extract (CER) isolated from Brazilian shrub *Solanum cernuum* leaves could chemosensitize T24 bladder cancer cells to cisplatin by decreasing MMP-2 or MMP-9 levels. Additionally, the results further showed that cotreatment of CER and cisplatin inhibited the phosphorylation and activation of p-ERK1/2, which is known to be associated with cancer cell survival, proliferation, and metastasis [163]. However, further study on *in vivo* and clinical trials is needed to confirm the chemosensitizing effect before it could be used as a complementary chemotherapy.

Cordycepin, a compound isolated and purified from *Cordyceps militaris*, also showed its potential use as a chemosensitizing agent towards temozolomide [164]. In the study conducted on glioma cells, cordycepin attenuated resistance against temozolomide by inhibiting the Akt pathway. Simultaneously, the expression levels of p-mTOR, p-p70S6K, and MMP-2 were reduced after treatment, with a lesser reduction extent for MMP-9. Because temozolomide is known to mediate the activation of Akt signaling that eventually leads to temozolomide resistance, thus, the combination of cordycepin and temozolomide reduced resistance by suppressing Akt signaling. Furthermore, downregulated MMP-2 and MMP-9 expression after cotreatment may explain the reduced cell migration and invasion observed [164].

### 4.3. Application of MMP-Responsive Nanomaterials for Targeting MMPs and Enhancing Chemotherapeutic Agent Delivery and Anticancer Activity

The inhibition of MMP activity in the extracellular space has been widely deliberated to inhibit the growth and invasion of cancer cells. Instead of targeting MMPs using MMPIs, taking advantage of their metastatic environment, the activation of drug carriers by MMP activity can ensure proper delivery in these highly metastatic sites. With innovations in nanomedicine, drug delivery systems that enhance the penetrability of drug molecules seem to gain traction in ensuring effective drug dissemination into tumor tissues. Several MMP-responsive nanomaterials have been effectively established, with the relevant information are summarized in Table 3.

Han et al. utilized MMP-2-responsive hyaluronic acid (HA) conjugated to poly (amidoamine) (PAMAM) carriers to deliver doxorubicin (DOX) into cancer cells [165]. This study utilized MMP-2 cleavage to dissociate the nanoparticle size from ~200 nm to its dendrimer units of size ~10 nm. The cleavage from MMP-2 activity not only enhanced nanoparticle extravasation and accumulation but also their retention, penetration, permeability, and diffusion. The *in vitro* cytotoxicity of doxorubicin was studied on two different cell lines, including MMP-2 high-expressing lung cancer A549 cells and MMP-2 low-expressing MCF-7 cells. In A549 cells, the cytotoxicity of HA-pep-PAMAM/DOX nanoparticles was not significantly different from that of MMP-2 pretreated HA-pep-PAMAM/DOX. This finding is due to that A549 cells expressing high MMP-2 levels to cleave the nanoparticles. Conversely, in low MMP-2 expressing MCF-7 cells, the cytotoxicity of MMP-2 pretreated HA-pep-PAMAM/DOX nanoparticles was significantly higher (IC_50_ = 0.760 *μ*g/mL) than that of HA-pep-PAMAM/DOX (IC_50_ = 1.884 *μ*g/mL) [165].

In another study, Nazli et al. [166] used a similar strategy to deliver DOX in an MMP-sensitive manner by inserting it into a MMP-degradable sequence conjugated with a PEG hydrogel coated with magnetic iron oxide nanoparticles (MIONPs). It was found that DOX particles were more efficiently delivered to the nuclei of cervical HeLa cells than free DOX. Intriguingly, MIONPs coated on the PEG hydrogel also enabled 11 times higher delivery rate than uncoated hydrogel nanoparticles. This study showed a promising use in improving the efficiency of existing chemotherapeutic drug delivery as well as minimizing drug resistance in cancer cells [166].

Furthermore, Dai et al. [167] synthesized a PEG-phosphoethanolamine (PEG-pp-PE) copolymer and successfully showed a reduction in drug resistance by Pgp activity in ovarian NCI/ADR-RES cells. The PEG-pp-PE copolymer was dependent on MMP-2 cleavage of the peptide linker (pp) to inhibit Pgp-mediated drug efflux. The copolymers with pp linker were also able to downregulate Pgp expression on the cell surface as compared to the expression in untreated NCI/ADR-RES cells (74% of untreated cells at 24 hours, 55% at 48 hours), while the copolymers without pp were less efficient (no change at 24 hours, 85% of untreated cells at 48 hours). The results from the cytotoxicity assay indicated increased cytotoxicity after treating with PEG-pp-PE with free paclitaxel (PTX) and PTX-loaded PEG-pp-PE as compared to free PTX treatment in Pgp expressing NCI/ADR-RES and MDA-MB-231 cells. However, in non-Pgp expressing A549 cells, the copolymers only became an obstacle in drug release due to micelle formation by copolymer units [167].

Two other studies conducted by Yao et al. [168, 169] utilized MMP-2 cleavable pp linkers for targeted drug delivery. In one study, they developed a dual-targeting micelle that is activated by MMP-2 and bound to folate receptors (FR) on the cell surface [168]. The MMP/FR micelle inhibited Pgp-mediated drug efflux on MMP-2 expressing and FR expressing ovarian NCI/ADR-RES cells and breast MDA-MB-231 cells. The polymeric micelles showed increased cytotoxicity as compared to the free drug administered, indicating that the micelles improved intracellular drug accumulation [168]. In the second study, the utilization of an “all-in-one” polymer-lipid conjugate (PEG2k-ppTAT-PEG1k-PE) was described [169]. This study utilized the trans-activating transcriptional activator (TAT) peptide to transport PTX molecules across the plasma membrane. In the study, the PEG2k acted as the outer shell, which was removed when MMP-2 cleaved the pp. TAT acts as the cell-penetrating middle layer that delivers the PEG1k-PE inner micelle core into the cell. In both studies, the micelles relied on MMP-2 activity for pp cleavage, which caused PEG deshielding and exposure of internal molecules responsible for cell entry. Besides, it was observed in these studies that cancer cells were sensitized to the drug due to the enhanced intracellular drug accumulation [168, 169].

Other studies involving nanocarriers have also shown to increase drug retention in cancer cells [169, 170]. The purpose of applying the MMP-responsive mechanism to these carriers is to improve drug delivery to metastatic sites of the tumor in which MMP activity is higher. Additionally, the MMP-responsive mechanism also allows for a more controlled drug release [171], hence allowing improved targeting.

### 4.4. Potential Biological Regulators of MMPs in Chemoresistance

Accumulating evidence has shown that biological molecules or signaling pathways can potentially regulate MMP expression that consequently affecting their chemosensitizing effects. Among which, tissue inhibitors, microRNAs (miRNAs), and epigenetic interactions are identified as the common regulators of MMPs, with their interactions and possible regulatory mechanism in regulating MMP expression and activity are discussed below.

#### 4.4.1. Tissue Inhibitors

General inhibitors like *α*2-macroglobulin, which is present in plasma and tissue, and specific inhibitors such as TIMPs block MMP activities. Four TIMPs have been identified in humans that are anchored in ECM or are extracellularly secreted, which bind noncovalently to MMPs in 1 : 1 ratio to form stoichiometric complexes [188]. A net decrease in TIMP level was seen to have a positive correlation of tumorigenesis [189], and its expression would be expected to reduce tumor progression. Thus, TIMP expression is a host-protective response [190]. Taking this into consideration, TIMPs were initially thought to be a potential candidate for therapeutic application in cancer, as they showed a good inhibitory effect on tumor growth in transgenic mouse model [191, 192]. However, their administration as protein structure and poor pharmacokinetics has limited their application in cancer therapy. Moreover, paradoxical effects of TIMPs, which have been reported to promote tumor cell growth in addition to inhibiting MMP activity, have further added to its difficulty [190].

TIMPs inhibit a broad spectrum of MMPs as well as disintegrin and ADAMs and A disintegrin and metalloproteinase with thrombospondin motifs (ADAMTSs). However, several studies have investigated the potential of altering TIMP expression to inhibit MMP activity. For instance, Escalona at el. revealed that a reduction in TIMP-2 expression enhanced chemosensitivity of ovarian cancer cell lines (e.g., FT282, JOSH2, and OVCAR4) towards cisplatin and paclitaxel [193]. Furthermore, *TIMP-2* silencing in OVCAR4 cells abolished elevated STAT3 phosphorylation induced by cisplatin and paclitaxel as compared to control OVCAR4 cells. It has been shown that STAT3 signaling can induce chemoresistance and CSC markers in response to chemotherapy [193]. This study suggested that TIMP-2 inhibition reduces chemoresistance, which may be induce by chemotherapeutic treatment as well as reduce existing chemo-resistant properties in cancer. Another study also discovered that platinum-resistant epithelial ovarian cancer cells showed TIMP-1 overexpression, which was regulated by MEK/ERK pathway [194]. Meanwhile, TIMP-3 overexpression was linked to inducing chemosensitivity towards cisplatin in laryngeal carcinoma [195]. Its upregulation was described to promote mitochondria-dependent apoptosis to reduce cisplatin resistance. Taken together, TIMP expression may be useful in gauging response to chemotherapeutic drugs as well as could be used as a mean of diagnosis and drug target to reduce chemoresistance by targeting MMP activity.

#### 4.4.2. microRNA Interactions with MMPs in Regulating Chemoresistance

microRNAs (miRNAs) are single-stranded noncoding small RNA of approximately 22 nucleotides that occur as a large family [196, 197]. Several miRNAs have been implicated in regulating MMP biological functions. As these functions are embedded in several processes that support cancer progression, such as angiogenesis, EMT, and ECM remodeling, thus, targeting the miRNA/MMP axis may have significant importance in treatment strategy to reduce chemoresistance [198]. Interactions between miRNAs and MMPs or their signaling pathways may affect or alter MMP activities that contribute to the emergence of drug resistance via cancer progression and EMT. However, studies involving miRNA and MMP interactions that directly affect chemoresistance have been limiting, as most investigations on their interaction are focused on inhibiting cell migration and metastasis. Although these studies are useful, the effects of their interactions on drug response and chemoresistance may need to be picked up. By studying their interactions and manipulating them, it may be possible to utilize them as a treatment strategy, both on its own or as an adjuvant treatment with other conventional anticancer drugs.

One such research involves miRNA miR-211 and MMP-9 in glioma. The miRNA was reported to be suppressed in high-grade glioma, and upregulation of miR-211 suppressed MMP-9 expression levels, which consequently reduced glioma cell invasion and migration. What makes this study interesting is that treatment of miR-211 and shRNA targeting MMP-9 together with temozolomide increased apoptotic DNA fragmentation in glioma CSCs as well as reduced drug efflux by Pgp, which is a well-known mechanism of chemoresistance [128].

#### 4.4.3. Epigenetic Interactions with MMPs in Chemoresistance

The contemporary epigenetic information proposes that there are discrete multilayered epigenetic mechanisms that mainly regulate MMPs, TIMPs, and collagen substrates [199]. However, DNA methylation and histone modification are the major indicators of epigenetic interactions. Genome-wide methylation profiling at base resolution establishes the genomic distribution of methylated sequences, which is called methylome. The individual cells have a unique methylome pattern compared to normal cells, which is altered in malignancies [200, 201]. Identifying specific methylome patterns can be useful in abrogating MMP overexpression. A recent study reported that epigenetic regulation of WNT2/*β*-catenin/MMP signaling could abrogate cancer growth, migration, and drug resistance [202]. Hypermethylation of the WNT2 promoter region was observed in ESCC cell lines, but no methylation was detected in normal esophageal epithelial cells, suggesting that it is possible to regulate MMP activity indirectly by regulating WNT2 expression. DNA methyltransferase inhibitor was used to reduce methylation in the WNT2 promoter regions that subsequently attenuated WNT2/*β*-catenin/MMP signaling [202].

Because epigenetic regulatory mechanisms remain widely unexplored in the context of MMP regulation or inhibition, it is possible that these methods may provide alternative strategies to induce chemosensitivity via MMP inhibition and regulation.

## 5. Conclusion

Chemoresistance poses a huge challenge in cancer treatment and management because it renders most available anticancer drugs ineffective. Overcoming chemoresistance may require a deep understanding of the mechanisms contributing to it. Of which, factors such as drug efflux mechanisms, the presence of CSCs, genetic and epigenetic mutations, and EMT may play a crucial role in contributing to the emergence of chemoresistance. The key role of MMPs in ECM degradation makes them an important player in EMT, hence contributing to chemoresistance mechanisms. Furthermore, their role has made them an attractive target for diagnostic and prognostic markers, as it is common that the expression levels of MMPs correlate to different stages of cancer and their possible outcome.

As discussed in this review, some MMPs are more prominently involved in inducing and regulating chemoresistance. Though some MMP activities cause chemoresistance directly, such as MMP-7, the general mechanism involves the progression of EMT and apoptosis inhibition. In addition to this, the members of the gelatinase family, particularly MMP-2 and MMP-9, seem to be the most involved in EMT-induced chemoresistance. Based on these mechanisms, MMPs are attractive targets for inducing chemosensitivity to enhance cancer management and utilizing their expression levels to determine disease severity in cancer patients.

In the past, several MMPIs have been developed, mainly to reduce cancer migration and invasion. Even so, these inhibitors did not make it into the market due to toxicity caused by poor selectivity and broad-spectrum activity. Several studies investigating MMP inhibition to reduce chemoresistance have been abundant, although no data on clinical activity has been reported. These studies on novel MMPIs, existing drugs, and phytochemical compounds have shown that it is possible to elicit chemosensitizing effects when combined with existing anticancer drugs. Furthermore, other strategies such as MMP-responsive drug delivery systems and induction of epigenetic interactions with MMPs have shown great potential to improve drug response in resistant cancers.

The whole purpose of reducing chemoresistance in tumors is to improve drug response and the need of high doses of drugs to elicit the same effect, hence reducing side effects and toxicities as well as minimizing the occurrences of relapses. With so much to work with, the most feasible strategy in the near future is via an adjuvant therapy approach, with an MMP inhibiting material and a chemotherapeutic drug. For future works, computational analysis in developing and identifying novel MMPIs can be a promising field of study. Besides, identification of interactions of MMPs with other molecules or regulators can open many more doors. The effects of noncoding RNAs such as miRNA, siRNA, and long noncoding RNA (lncRNA) on MMP expression can be explored. For instance, lncRNAs such as *UCA1* and *HOTTIP* have been implicated to play a promising role in carcinogenesis, cancer progression, and chemoresistance [203, 205]. Thus, further exploration into these topics can be done to discover potential therapeutic targets or diagnostic or prognostic markers.

Studies on MMPs and their role in cancer have been conducted for decades. However, only recently their role in chemoresistance started to gain attention. Based on this review, it has provided insights that MMPs can induce and regulate chemoresistance in different cancers via the interactions with different molecules and signaling pathways. Nonetheless, more investigations are still required to fully utilize the knowledge effectively. Furthermore, by expanding the knowledge in this field, increasing alternative strategies can be developed and worked on from existing knowledge.

## Figures and Tables

**Table 1:** Roles and mechanisms of specific MMPs commonly observed in chemoresistance.

MMP	Target/signaling pathway/interaction	Biological effect
MMP-1	Promoter hypomethylation	Tamoxifen resistance
	Overexpression of TS and DPD	Resistance to 5-FU
	Interaction with CAF	Cetuximab resistance, Taxotere resistance
	Interaction with slug, increase EMT marker expression	Adriamycin resistance
	Interaction with HOXA9 and PBX1, overexpression of COPS5	Erlotinib resistance
MMP-2	NFE2L2	Drug resistance via apoptosis inhibition
	Upregulation of orosmucoid 1, Akt/ERK pathway	Epirubicin resistance
	MAPK pathway	Vemurafenib resistance
	*Β*-catenin, p-c-raf	Paclitaxel resistance
	ERK1/2 pathway	Adriamycin resistance
MMP-7		
	Fas/FasL	Oxaliplatin, doxorubicin, docetaxel, cisplatin resistance
	IGFBP-3	Resistance to drugs such as anthracycline and taxane
	Syndecan-1 cleavage, EGFR phosphorylation	5-FU, oxaliplatin, irintecan, cisplatin, or paclitaxel resistance
MMP-9	Induction of IKK activity	Resistance to 5-FU
	Increased Pgp efflux	Increased drug efflux from cancer cells
	Increased expression of VEGF	Oxaliplatin resistance
MMP-14	Interaction with CD133^+^ cells	Increased MMP-14 expression is common in CD133+ cells, which leads to CSCs formation and drug resistance against cisplatin and paclitaxel
	Phosphorylation of *γ*H2AX	Resistance against doxorubicin
	Phosphorylation of ERK1/2 and HMGA2	Resistance against gemcitabine

**Table 2:** Clinical trials conducted on MMP inhibitors and their status.

Name of inhibitor	Type of inhibitor	MMPs targeted	Chemotherapeutic agent	Type of cancer studied	Toxicity	Outcome	Ref/NCT
AB0041, AB0046, GS-5745	Monoclonal antibody	MMP-9	NA	Colorectal	NA	Active in preclinical studies	[151]
Andecaliximab (GS-5745)	Monoclonal antibody	MMP-9	NA	Gastric, breast, pancreatic, nonsmall cell lung, esophageal, colorectal	Neutropenia, nausea, pain, GI upset	Ongoing phase I, II, and III clinical trials	NCT02862535, NCT02864381, NCT02545504
Batimastat (BB-94) 5362422∗	Hydroxymate (zinc chelator)	Broad, including MMP-1, -2, -3, -7, -9, -14	Temozolomide	Malignant ascites (pancreatic, colorectal, gastric, ovarian, cholangiocarcinoma, ovarian, mesothelioma) Malignant pleural effusion (nonsmall cell lung, breast, melanoma, renal, mesothelioma)	Musculoskeletal syndrome, fever, liver function abnormalities, pleural pain at the site of injection	Cancelled in phase III clinical trials (local toxicity, slow accrual, Marimastat developed)	[148, 149, 152]
DX-2400	Monoclonal antibody	MMP-14	NA	Breast, melanoma, fibrosarcoma	NA	Active in preclinical studies	[153]
Marimastat (BB-2516) 119031∗	Hydroxymate (zinc chelator)	Broad, including MMP-1, -2, -3, -7, -9	Paclitaxel, carboplatin	Breast, nonsmall cell lung, colorectal, pancreatic, gastric, prostate, glioblastoma	Musculoskeletal syndrome, GI upset	Prolongation of survival in randomized Ph2 in gastric cancer, cancelled in phase III clinical trials	NCT00003010, NCT00002911 [154]
Prinomastat (AG3340) 466151∗	Hydroxymate (zinc chelator)	MMP-2, -3, -9, -13, -14	Carboplatin	Nonsmall cell lung, esophageal	Musculoskeletal, venous thromboembolism, hematologic, GI upset	Cancelled in phase III clinical trials	NCT00004200, NCT00004199, NCT00003343
Rebimastat (BMS-275291) 9913881a	Sulfhydryl-based mercaptoacyl (zinc chelator)	MMP-1, -2, -3, -8, -9, -13, -14	Paclitaxel, carboplatin	Nonsmall cell lung, breast, prostate	Increased toxicity, dermatologic hypersensitivity	Cancelled in phase III clinical trials	NCT00040755, NCT00039104, NCT00006229 [155]
Tanomastat (BAY 12-9566) 6918336a∗	Carboxylate (zinc chelator)	MMP-2, -3, -8, -9, -13	Paclitaxel	Pancreatic, ovarian, small cell lung	Hematologic (anemia, thrombocytopenia), electrolyte abnormalities, hyperbilirubinemia, GI upset	Cancelled in phase III clinical trials	[156–158]
MMP-9/MMP-2 inhibitor ((2*R*)-2-[(4-biphenylsulfonyl) amino]-3 phenylpropionic acid	Synthetic inhibitor	MMP-9/MMP-2	Cisplatin	Ovarian	NA	Only *in vitro* studies	[132]
Metformin	Type 2 diabetes drug	MMP-9, uPA	Sorafenib	Hepatocellular carcinoma	NA	Only *in vitro* studies	[150]

∗PubChem identification number.

**Table 3:** Typical MMP-responsive nanocarriers for delivery of anticancer drugs.

Nanocarriers	Functional nanomaterials	Anticancer drug	Cancer	Biological effect	Ref
Macromolecule-based conjugates	Polymer-peptide-drug conjugates	Methotrexate	Fibrosarcoma, glioblastoma, bladder carcinoma	Dextran-PVGLIG-methotrexate conjugates: prolonged blood circulation; improved tumor targeting and anticancer activity; decreased side effects	[172]
		Doxorubicin	Lewis lung carcinoma	PEG-peptide-DOX conjugates: self-assembly to micelles; MMP2-dependent cytotoxicity; tumor growth inhibition. Peptide, GPLGV, or GPLGVRG	[170]
		Doxorubicin	Colon, breast	PEG-ppTAT-DOX conjugates: self-assembly to nanoparticles; MMP2-dependent cell penetration and cytotoxicity; drug efflux inhibition	[173]
		Paclitaxel	Nonsmall cell lung cancer	PEG2k-pp-PTX conjugates: self-assembly; MMP2-dependent uptake, penetration, and cytotoxicity; improved tumor targeting and anticancer activity	[174]
	Albumin-peptide-drug conjugates	Doxorubicin	Renal	DOX albumin conjugates: MMP2/9-dependent cytotoxicity	[175]
		Doxorubicin	Melanoma	DOX albumin conjugates: MMP2-sensitive drug release; improved in vivo anticancer activity and decreased adverse effects	[176]
Liposomes	Polymer-lipid conjugates	N4-Octadecyl-1-*β*-D-arabinofuranosylcytosine (NOAC), lipophilic derivative of ara-C	Hepatocellular carcinoma	PEG-pp-PE-modified galactosylated liposomes: MMP2-triggered PEG deshielding; MMP2-responsive cellular uptake and cytotoxicity.	[177]
	MMP-sensitive triple helical peptides	—	Melanoma, hepatocellular carcinoma	“Uncorking” liposomes: MMP9-triggered liposomal “uncorking” and cargo release	[172–174]
Micelles	Polymer-peptide conjugates	Doxorubicin	Fibrosarcoma, breast, ovarian	Phenylacetyl-peptide micelles: MMP9-dependent morphological change from micelles to nanofibers; enhanced anticancer activity	[165, 175]
	Polymer-lipid conjugates	Paclitaxel	Ovarian	PEG-pp-PE micelles: MMP2-dependent particle size, drug release, and cytotoxicity; reversal of multidrug resistance	[167]
		Paclitaxel	Fibrosarcoma, breast, ovarian, nonsmall cell lung cancer	MMP2-sensitive CPP-modified micelles: MMP2-dependent cellular uptake and anticancer activity; reversal of multidrug resistance	[178]
		Dasatinib	Ovarian, breast	MMP2 and FR dual-targeted micelles: MMP2-dependent uptake, penetration and anticancer activity; improved PK, biodistribution, and tumor targeting	[168]
		Paclitaxel	Fibrosarcoma, breast, ovarian	All-in-one micelles (PEG2k-ppTAT-PEG1k-PE): high stability; MMP2-responsive cellular uptake and penetration; improved tumor retention	[174]
	PEG-peptide-cationic polymer conjugates	—	Cervical	PEG-GPLGVRG-PAsp (DET) polyplex micelles: MMP2-responsive cellular uptake and endosomal escape; improved gene transfection	[179]
		Paclitaxel, siRNA	Lung	PEG2k-pp-PEI-PE micelles: drug and siRNA codelivery; MMP2-responsive charge conversion; improved uptake, gene silencing, and anticancer activity	[180]
Protein nanoparticles	Gelatin nanoparticles	Doxorubicin	Colon, breast	QDs-loaded gelatin nanoparticles: MMP2-responsive gelatin degradation and QD release; tumor targeting and deep tumor penetration	[173]
		Doxorubicin	Breast	Dendrimer-loaded gelatin nanoparticles: MMP2-responsive degradation and dendrimer release; size-dependent tumor targeting and tissue penetration	[181]
		Doxorubicin	Glioma	AuNP-loaded gelatin nanoparticles: MMP2-responsive gelatin degradation and AuNP release; improved tumor targeting and imaging	[182]
Polymeric nanoparticles	Activatable protamine	Doxorubicin	Glioblastoma	ALMWP-conjugated PEG-PCL nanoparticles: MMP2/9-dependent cellular uptake and cytotoxicity; enhanced tumor targeting and anticancer activity	[183]
	MMP-sensitive polypeptide	Paclitaxel	Lung	LinTT1-PVGLIG-TAT-modified PEG-PLA nanoparticles: MMP-responsive cellular uptake; improved tumor targeting and anticancer activity	[184]
	PEG-peptide-PLA	Paclitaxel	Breast, liver	PEG-GPLGVRGDG-PLA nanoparticles: MMP2-responsive PEG deshielding and RGD exposure; improved tumor targeting and anticancer activity	[185]
Dendrimers	MMP-sensitive peptides	Doxorubicin	Lung	HA-PLGLAG-poly(amidoamine) dendrimers: MMP2-dependent size shrinkage; improved tumor targeting and anticancer activity	[165]
Nanogels	MMP-sensitive proteins or peptides	Doxorubicin	Cervical	Polypeptide-based crosslinked hydrogels: nanogel formation via electrostatic interaction; MMP9-dependent gel destabilization and cargo release	[186]
		Doxorubicin	Breast	Dendrimer/collagen hybrid gels: MMP-sensitive cytotoxicity; suppression of tumor growth and metastasis *in vivo*	[187]
Inorganic nanoparticles	MMP-sensitive iron oxide nanoparticles	Doxorubicin	Cervical	PEG-coated magnetic iron oxide nanoparticles: MMP-dependent PEG deshielding and cellular uptake; improved intracellular drug release	[166]

## Data Availability

The data supporting this manuscript are extracted from the previously reported studies and data sets, which have all been cited.
